# Self-care and lifestyle interventions of complementary and integrative medicine during the COVID-19 pandemic—A cross-sectional study

**DOI:** 10.3389/fmed.2022.1033181

**Published:** 2023-01-09

**Authors:** Michael Jeitler, Avital Erehman, Daniela A. Koppold, Miriam Ortiz, Lea Jerzynski, Barbara Stöckigt, Gabriele Rotter, Sarah Blakeslee, Benno Brinkhaus, Andreas Michalsen, Georg Seifert, Holger Cramer, Farid I. Kandil, Christian S. Kessler

**Affiliations:** ^1^Charité – Universitätsmedizin Berlin, Corporate Member of Freie Universität Berlin and Humboldt-Universität zu Berlin, Institute of Social Medicine, Epidemiology and Health Economics, Berlin, Germany; ^2^Department of Internal and Integrative Medicine, Immanuel Krankenhaus Berlin, Berlin, Germany; ^3^Department of Paediatric Oncology/Haematology, Otto-Heubner Centre for Paediatric and Adolescent Medicine (OHC), Charité – Universitätsmedizin Berlin, Corporate Member of Freie Universität Berlin and Humboldt-Universität zu Berlin, Berlin, Germany; ^4^Departamento de Pediatria, Faculdade de Medicina, Instituto de Tratamento do Câncer Infatil (ITACI), Universidade de São Paulo, São Paulo, Brazil; ^5^Department of Internal and Integrative Medicine, Evang. Kliniken Essen-Mitte, Faculty of Medicine, University of Duisburg-Essen, Essen, Germany; ^6^Institute for General Practice and Interprofessional Care, University Hospital Tuebingen, Tübingen, Germany; ^7^Bosch Health Campus, Stuttgart, Germany; ^8^National Centre for Naturopathic Medicine, Southern Cross University, Lismore, NSW, Australia

**Keywords:** self-care, lifestyle medicine, prevention, COVID-19 pandemic, complementary medicine, integrative medicine, infectious respiratory diseases

## Abstract

**Background:**

Complementary and Integrative Medicine (CIM), including self-care healthy life-style promotion strategies, is widely used in Germany. Aim of this study was to assess the use of self-care and lifestyle interventions as well as mental/emotional state experienced during the COVID-19 pandemic.

**Methods:**

An exploratory cross-sectional online study was conducted with adults in Germany through an online survey. Custom-developed questions in respiratory disease-status (including COVID-19), CIM-based self-care/lifestyle interventions and dietary patterns, and mental/emotional state as well as parameters for wellbeing (World Health Organization Well-Being Index, WHO-5) and self-efficacy (General Self-Efficacy Short Scale, GSE-3) were assessed. Data was analyzed using frequency and parametric measures.

**Results:**

The online survey was performed from January to March 2021 and included 1,138 participants (81.5% female; mean age: 49.2 ± 13.7 years; 54.9% holding a university degree) living in Germany, of which 62 had had a positive SARS-CoV-2 test, 4 an influenza infection and 375 participants other respiratory infections. The following individual health promotion strategies were reported: spending time in nature (90%; *n* = 1,024), physical activity (69.3%; *n* = 789), naturopathic remedies (63.1%; *n* = 718), plant-based diet (56.3%; *n* = 640), and Mind-Body interventions (54.7%; *n* = 623). No differences in strategies between individuals with respiratory diseases or the sample overall were found. Well-being had a mean value of 15.2 ± 5 (WHO-5) and self-efficacy 4.1 ± 0.6 (GSE-3). Nearly 8% reported a low mental/emotional state regarding the COVID-19 pandemic.

**Conclusion:**

Self-care and lifestyle interventions during the COVID-19 pandemic were reported by participants who were predominantly female, middle-aged, and well-educated. Most participants showed an overall balanced mental/emotional state. Further studies should include a representative control group from the general population.

**Clinical trial registration:**

clinicaltrials.gov, identifier NCT04653727.

## 1. Introduction

With the ongoing coronavirus disease 2019 (COVID-19) pandemic, humanity still faces a global health threat: by the end of August 2022, more than 600 million infections and 6 million deaths worldwide are expected to be related to severe acute respiratory syndrome coronavirus 2 (SARS-CoV-2) (1). The COVID-19 pandemic, as well as previous viral epidemics or world-wide pandemics, will most likely continue to threaten health systems, societies, and economies worldwide (2, 3).

Evidence-based Complementary and Integrative Medicine (CIM) interventions, such as Mind-Body Medicine (MBM), herbal therapies, and nutritional medicine, are increasingly used in Germany, Europe and worldwide and have the potential to provide personally-tailored complementary medical strategies as part of an optimized overall health care management (4–6). CIM offers a variety of preventive and therapeutic options for strengthening physical and mental resilience that may be useful during the COVID-19 pandemic and beyond (7).

The multifold relationships between the immune system and a variety of health-increasing lifestyle factors such as exercise, stress reduction, healthy diet, spending time outdoors, maintaining a positive attitude, and preserving wellbeing have been demonstrated in various studies (8–15). Thus, CIM interventions have the potential to be used to improve immune functions and enhance quality of life and wellbeing in the COVID-19 pandemic, which caused stress, anxiety, fear, and depression in many individuals and societies around the world (16).

Global recommendations on how to stay healthy during the COVID-19 pandemic from authorities and health professionals, refer to a healthy lifestyle in addition to appropriate hygiene and social measures. Sufficient sleep, healthy diet including ample consumption of fresh fruit and vegetables, stress reduction, and staying active are examples of such measures recommended by the World Health Organization, yet are insufficiently studied (17).

Cross-sectional surveys have been conducted at different time points during the pandemic. Physical activity, nature stays and MBM-interventions such as yoga, meditation, and relaxation techniques were the most frequently used health-promoting interventions (18–20). However, the extent of the use of such self-care interventions among CIM users in Germany during the pandemic remains largely unclear.

The aim of this cross-sectional study was to investigate the extent of CIM self-care and lifestyle interventions use and their associations with infectious respiratory diseases including COVID-19 and assess the mental/emotional state during the first and second wave of the COVID-19 pandemic in Germany.

## 2. Materials and methods

### 2.1. Study design and setting

This explorative cross-sectional study was conducted between January 6th, 2021 and March 5th, 2021. People were asked to participate *via* an anonymous online survey in German, English, Spanish, or Portuguese language. The study was conducted by the Charité Outpatient Department for Complementary and Integrative Medicine at Immanuel Hospital Berlin and the Institute of Social Medicine, Epidemiology and Health Economics of the Charité – Universitätsmedizin Berlin. The study was approved by the Charité – Universitätsmedizin Berlin Ethics Committee (EA1/187/20) and registered at ClinicalTrials.gov (NCT04653727).

### 2.2. Participants and recruitment

Participation required internet access. Participants were recruited primarily through social media (e.g., Twitter, Facebook, websites, and online newsletters) and *via* the following non-profit associations “Kneipp-Bund e.V.” (Kneipp association), “Natur und Medizin e.V.” (Nature and Medicine), “Gesundheit aktiv e.V.” (active health), “ProVeg Deutschland e.V.” (ProVeg Germany), and printed flyers in the Charité Outpatient Department for Complementary and Integrative Medicine at Immanuel Hospital Berlin. The questionnaire was aimed at adults who considered themselves to have an affinity for CIM and/or lifestyle interventions. Prior to participation, each participant was asked to provide informed consent by checking a box on the digital platform, and the participant’s age was verified.

### 2.3. Outcome measurement and data collection

The questionnaire was implemented using Limesurvey (LimeSurvey GmbH, Hamburg, Germany, version 4) on a Charité server. Depending on the question content, answers enabled either single or multiple responses. The estimated time to complete the questionnaire was around 30 min.

Sociodemographic data including age, gender, household size, school education, employment status, and monthly net income was collected. Participants were asked whether COVID-19, influenza, and other respiratory infections had occurred since March 2020, and if so, asked to give additional detail on symptom severity and hospitalization. Moreover, risk factors for a severe course of COVID-19 were asked. Using a custom questionnaire, a selection of general health-related lifestyle factors and duration of their use since March 2020 were queried, including dietary habits, sports activity and CIM interventions such as time spent in nature, individual use of hydrotherapy/Kneipp applications, anthroposophical medicine, intermittent and/or periodic and therapeutic fasting, botanical/herbal remedies, and Mind-Body interventions. Also, illness-related lifestyle behavior was asked about including alcohol consumption, tobacco consumption, and sedentary behavior. Additional interventions could be entered in an open-ended free text field after choosing “other.” Validated questionnaires assessed the current self-efficacy with a 3-item questionnaire General Self-Efficacy Short Scale (GSE-3), the German-language scale is called Allgemeine Selbstwirksamkeit Kurzskala (ASKU), and well-being within the last 2 weeks with the 5-item World Health Organization Well-Being Index (WHO-5) (21, 22). To measure the mental/emotional state since March 2020, eight custom-developed questions with numerical rating scales (NRS; 0–10 points; 0 = minimum to 10 = maximum) were used. These eight items included distress, anxiety, depression, exhaustion caused by COVID-19 pandemic, fear of being infected with SARS-CoV-2, fear of financial/economic consequences, fear of negative societal consequences with referred examples of loneliness, or increase in social inequality, and sleep quality. The values of the eight items related to the mental/emotional state were added to a total sum score ranging from 0 to 80 points. Participants that had a calculated 0–26 points were defined as having a “positive mental/emotional state” whereas those with a calculated 54–80 points were defined as having a “negative mental/emotional state.” In subgroup analysis participants with a calculated positive mental and emotional state were compared to those with a negative mental/emotional state to investigate how mental/emotional state is related to specific CIM interventions. Further subgroup analyses compared gender (male and female) and age categories (18–30 years, 31–50 years, 51–65 years, and ≥66 years old).

As an incentive for survey participation, participants had the option in the anonymized survey to provide their email address in order to enter a lottery to win one of 20 books about CIM that were drawn and distributed the end of the study in March 2021.

### 2.4. Statistical analysis

Descriptive statistical analysis was carried out with both International Business Machines Corporation (IBM) SPSS Statistics (version 26) and Python (version 3.7). Given the explorative nature of the study, no sample size calculation was performed. We initially aimed to include 3,000 participants.

Data were analyzed with descriptive statistics first for the whole group for absolute and relative frequencies (numbers and percent), for observed numbers, and for mean and standard deviation (“M” and “SD”). Subsequent subgroup-analysis was conducted for a number of predefined factors, including gender (female vs. male participants); age group (18–30, 31–50, 51–65, and ≥66 years of age); experienced infection with COVID-19, influenza or any other respiratory infection during March 2020 (with yes-no options for each factor); and lastly, given the calculated positive vs. negative mental/emotional state in our custom-written questionnaire (see above). Due to the exploratory nature of the study, statistical hypothesis tests were not conducted.

## 3. Results

### 3.1. Sociodemographic data

This exploratory cross-sectional online-study was launched on January 6th, 2021 and was accessible online for 2 months. A total of 1,563 people consented and participated in the survey. This survey was conducted as an international survey in four different languages (German, English, Spanish, and Portuguese). A total of 1,287 participants completed the survey. To keep the study population as homogeneous as possible, we decided to report only the *n* = 1,138 (96%) data sets of survey-participants who lived in Germany since March 2020 in this publication. Of these, 1,134 participants completed the questionnaire in German language, three in English, and one in Spanish. Apart from these, 57 complete data sets from Brazil, 30 complete data sets from Austria, 22 complete data sets from Switzerland, 7 complete data sets from Spain, and 3 data sets each from Portugal and United Kingdom and 31 data sets from other countries, which are not reported in detail in the following, were obtained. *N* = 277 started the survey without completing it, or even beginning to answer the first question, and were thus excluded from the analysis. Recruitment was discontinued due to a sharp decrease in responses on March 5th, 2021.

All in all, the 1,138 datasets from the German were included in the final descriptive analysis in this paper.

Participants were mainly female (81.5%; *n* = 927), middle-aged (49.2 ± 13.7 years), had a high level of average income and education (Table 1). There are four age groups described in this publication, which were distributed as follows: 11.6%; *n* = 132 were 18–30 years old, 35.4%; *n* = 403 were 31–50 years old, 43.2%; *n* = 492 were 51–65 years old and 9.8%; *n* = 111 were ≥66 years old. More than half of the participants (54.9%; *n* = 625) had a university degree. Most participants worked full-time (more than 35 h/week) (36.9%; *n* = 420), while 26.7% (*n* = 304) worked part-time (15–34 h/week). Retirement was reported by 11.7% (*n* = 133) of participants. Nearly half of the participants were married (44.5%; *n* = 529) and an additional 23.1% (*n* = 263) were in a relationship (Table 1).

**TABLE 1:** Sociodemographic data.

				Gender			
		All		Female		Male	
		*N*	%	*N*	%	*N*	%
Age	(Mean ± SD)	49.16 ± 13.7		49.07 ± 13.33		49.74 ± 15.3	
Gender	Male	207	18.2	0	0	207	100
	Female	927	81.5	927	100	0	0
	Diverse	4	0.4	0	0	0	0
Family status	In relationship	263	23.1	214	23.1	48	23.2
	Married	529	46.5	423	45.6	106	51.2
	Single	198	17.4	155	16.7	41	19.8
	Divorced	93	8.2	86	9.3	6	2.9
	Widowed	24	2.1	22	2.4	2	1
	Not specified	31	2.7	27	2.9	4	1.9
Adults in household	(Mean ± SD)	2.47 ± 7.97^1^		2.32 ± 7.75^1^		3.12 ± 8.92^1^	
Children in household	(Mean ± SD)	0.58 ± 2.33		0.51 ± 1.71		0.94 ± 4.07^1^	
Monthly net income	<1,000 €	176	15.5	154	16.6	21	10.1
	1,001–1,500 €	135	11.9	118	12.7	16	7.7
	1,501–2,000 €	198	17.4	170	18.3	28	13.5
	2,001–3,000 €	206	18.1	153	16.5	53	25.6
	3,001–4,000 €	116	10.2	83	9	33	15.9
	>4,000 €	84	7.4	56	6	27	13
	Not specified	223	19.6	193	20.8	29	14
Monthly net income for entire household	<1,500 € $	86	7.6	72	7.8	12	5.8
	1,501–2,000 €	106	9.3	93	10	13	6.3
	2,001–3,000 €	164	14.4	130	14	34	16.4
	3,001–4,500 €	224	19.7	174	18.8	50	24.2
	4,501–6,000 €	142	12.5	110	11.9	32	15.5
	>6,001 €	122	10.7	97	10.5	24	11.6
	Not specified	294	25.8	251	27.1	42	20.3
Highest educational qualification	University degree	625	54.9	507	54.7	117	56.5
	Completed training in an apprenticeable trade	176	15.5	145	15.6	29	14
	Higher education entrance qualification (A-level)	202	17.8	163	17.6	39	18.8
	Intermediate school leaving certificate or secondary school leaving certificate	93	8.2	79	8.5	14	6.8
	Secondary school diploma	16	1.4	10	1.1	6	2.9
	Elementary School Certificate	2	0.2	2	0.2	0	0
	No school-leaving qualification yet (pupil)	24	2.1	21	2.3	2	1
Current employment	Full time (min 35 h/week)	420	36.9	318	34.3	101	48.8
	Part-time (15–34 h/week)	304	26.7	264	28.5	40	19.3
	By the hour (under 14 h/week)	72	6.3	65	7	7	3.4
	Training/study	53	4.7	44	4.7	9	4.3
	Maternity/parental leave	21	1.8	20	2.2	1	0.5
	Long-term sick leave (>4 weeks)	16	1.4	14	1.5	1	0.5
	Retired	133	11.7	104	11.2	29	14
	Unemployed with social benefits	26	2.3	22	2.4	4	1.9
	Working with social benefits	9	0.8	8	0.9	1	0.5
	Not specified	84	7.4	68	7.3	14	6.8

Tables report *n* and % (if not specified). ^1^*n* = 5 live in “extended” families between 5 and 40 children.

### 3.2. Chronic diseases, alcohol/cigarette use and sick leave

The mean Body mass index (BMI) was 23.9 ± 6.3 kg/m^2^, 7% (*n* = 83) were obese (BMI ≥ 30 kg/m^2^) and 3% (*n* = 36) were underweight (<18.5 kg/m^2^) (Supplementary Table 1). Nine percent of participants (*n* = 116) listed a diagnosis of chronic cardiovascular disease (most frequently high blood pressure with *n* = 82; 7.2% of participants) and 10.3% (*n* = 132) a chronic respiratory disease (most frequently bronchial asthma with *n* = 70; 6.2% participants), see Supplementary Table 1. Consumption of alcohol had a mean of 6.4 ± 7.7 units per week for 33% (*n* = 379) of the participants. Men consumed twice as many units of alcohol (10.9 ± 12.1) as women (5.3 ± 5.8) and those who had negative mental/emotional state consumed more alcohol (average 8.7 ± 6.5 units) than those with positive mental/emotional state (average 6.9 ± 6.1 units; one alcoholic unit meant 0.25 L beer, 0.1 L wine, 0.1 L sparkling wine, or 0.04 L spirits). Cigarette use averaged 7.8 ± 6.4 cigarettes, with men using slightly more (9.1 ± 7.7) than women (7.4 ± 6 cigarettes per week).

Nearly a quarter, 22.8% (*n* = 259), had taken sick leave since March 2020 (8.9 ± 39.5 days), and those who had a negative mental/emotional state (19.9 ± 58.8 days) and who tested positive for SARS-CoV-2 (15.3 ± 39.2 days) having had longer sick leaves.

### 3.3. Affectedness by COVID-19

Positive testing for SARS-CoV-2 was reported by 5.4% (*n* = 62) (Supplementary Table 2). Only a single participant (0.9%; *n* = 1) had tested positive for SARS-CoV-2 in the age group ≥66 years (of all *n* = 111 in this age group). One percent, *n* = 11, of those who had tested positive the SARS-CoV-2 reported being symptom-free again and 4.5%; *n* = 51 continued to have symptoms of COVID-19. Mainly mild or moderate symptoms were described with a symptom duration of 19.1 ± 23.3 days. The symptom with the highest described impact was “exhaustion” with 6.7 ± 2.7 (on a NRS; 0–10 points; 0 = minimum to 10 = maximum). None of those who described symptoms reported needing hospitalization or in intensive care treatment. Twenty-nine participants (46.8% of those tested positive for SARS-CoV-2) reported persisting Post-COVID symptoms with a moderate symptom severity (3.9 ± 3.9) (NRS 0–10).

Since March 2020, those who reported a COVID-19 diagnosis regularly used various CIM self-care and lifestyle interventions, such as spending time outdoors (91.9%; *n* = 57), exercise (74.2%; *n* = 46), MBM interventions (51.6%; *n* = 32), fasting (46.8%; *n* = 29), anthroposophical medical applications (3.2%; *n* = 2), hydrotherapy or water treatments (16.1%; *n* = 10), nasal rinses (11.3%; *n* = 7), naturopathic remedies (see below) (53.2%; *n* = 33), digital health services (30.6%; *n* = 19), and aromatherapy (*n* = 6; 9.7%). More than half of all participants who had tested corona-positive had used naturopathic remedies as well as dietary supplements, particularly vitamin D (33.9%; *n* = 21), vitamin B complex or vitamin B12 (27.4%; *n* = 17) and magnesium (9.7%; *n* = 6).

Views about restrictions during the pandemic split the sample into approximate thirds: 38.4% of all participants (*n* = 437) felt that the restrictions during the pandemic were just right, while 34.7% (*n* = 395) thought they were excessive and 26.9% (*n* = 306) thought they should be tougher. Vaccinations on the other hand skewed toward resistance to being vaccinated: 32.5% of all participants (*n* = 370) planned to get vaccinated (25%; *n* = 285 might/40.2%; *n* = 458 would not). In terms of adhering general hygiene regulations (distance, mask, etc.) starting March 2020, participants who had been COVID-19 positive were similarly compliant (8.18 ± 2.25) to the sample overall (8.09 ± 2.29) (NRS 0–10).

### 3.4. Effect of influenza and other infectious respiratory diseases

A total of 33% of participants (*n* = 375) had had other infectious respiratory diseases since March 2020 (Supplementary Table 3). These respondents had predominantly experienced moderate symptoms for an average duration of 12.5 ± 19.2 days. Only 0.4% of participants (*n* = 4) had been infected with influenza. All of them reported experiencing severe symptoms (8.0 ± 1.8 on a 0–10 NRS), with none being hospitalized or treated in intensive care. Those who reported being affected by influenza did not show major differences from the overall sample in terms of CIM self-care and lifestyle interventions. A total of 17.3% of all participants (*n* = 197) had received the seasonal flu vaccine.

A slightly larger proportion of the participants suffering from other respiratory diseases (8.5%; *n* = 32) were in a negative mental/emotional state compared to the overall sample (7.6%; *n* = 87). In comparison to the overall sample, participants who reported having been infected with other respiratory diseases, were shown to have slightly higher average levels of psychological stress parameters (NRS 0–10) during the pandemic like feeling stressed about the SARS-CoV-2 pandemic (4.9 ± 2.7 vs. 4.4 ± 2.7), anxious (3.2 ± 2.6 vs. 2.7 ± 2.5), depressed (3.5 ± 2.8 vs. 3 ± 2.8), exhausted (4.3 ± 2.8 vs. 4 ± 2.8), fear of being infected by SARS-CoV-2 (2.8 ± 2.5 vs. 2.4 ± 2.4), and had a lower sleep quality (4.2 ± 2.7 vs. 3.8 ± 2.7).

### 3.5. Use of self-care/lifestyle and CIM interventions during the COVID-19 pandemic

**FIGURE 1:**
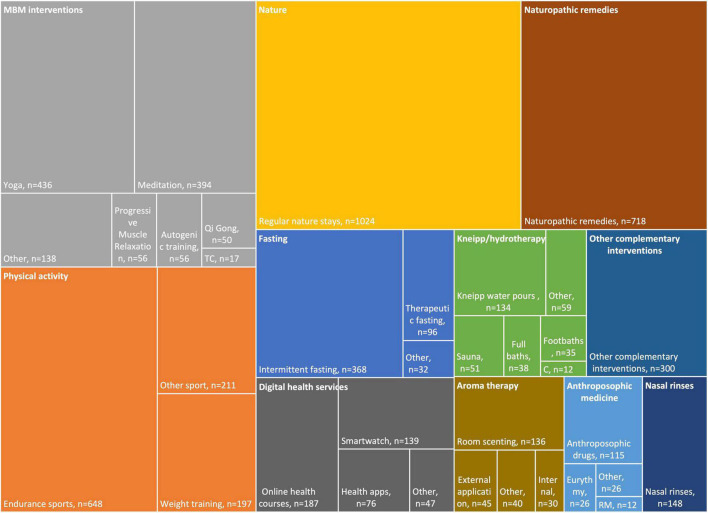
Treemap on Complementary and Integrative Medicine (CIM) self-care interventions during the COVID-19 pandemic, sorted by main topic (upper left corner). *C*, Compresses; MBM, Mind-Body Medicine; RM, Rhythmical Massage; TC, Tai Chi.

Starting in March 2020, respondents described various self-care and lifestyle interventions, with a preference for spending time outdoors (90%; *n* = 1,024), practicing physical activity (69.3%; *n* = 789), using naturopathic remedies (63.1%; *n* = 718), and undertaking MBM activities (54.7%; *n* = 623) (see Supplementary Table 4 and Figure 1). Other interventions such as intermittent fasting (32.3%; *n* = 368), digital health services (27.5%; *n* = 313), Kneipp/hydrotherapy (17%; *n* = 193), aromatherapy (13.4%; *n* = 153), and anthroposophical medicine (11.6%; *n* = 132) were also used. Yoga and meditation were the most frequently practiced MBM interventions (38.3%; *n* = 436 and 34.6%; *n* = 394, respectively). For other listed interventions reported see Supplementary Table 4.

Complementary and Integrative Medicine methods were used more frequently by women than by men, e.g., naturopathic remedies (+16.2%), MBM interventions (+13.7%), digital health applications (+12.9%), and aromatherapy (+11.6%). Differences were also found in the age categories. Thus, participants from the older age category (≥66 years old) reported using anthroposophical medicine applications (+13.8%) and hydrotherapy or water treatments (+15.4%) more frequently and less frequently used physical activity (−18.8%) and digital health applications (−20.3%) than younger participants (18–30 years old). Moreover, participants in a positive mental/emotional state used MBM techniques (+13%) more often than those in a negative emotional state, which was accompanied by differences in practice duration of MBM. Respondents in a positive mental/emotional state demonstrated a longer practice duration of yoga (144 ± 142.2 vs. 104.8 ± 115.2 min/week), meditation (148.2 ± 171.6 vs. 88.6 ± 62 min/week), Tai Chi (52.8 ± 44.3 vs. 33.3 ± 23.1 min/week), and progressive muscle relaxation (44.3 ± 39.2 vs. 40 ± 35.8 min/week), compared to respondents in a negative emotional state who had a longer practice of Qi Gong (74.8 ± 69.3 vs. 130 ± 180.7 min/week) and autogenic training (50.3 ± 44.9 vs. 61 ± 40.7 min/week).

For participants in a positive emotional state, longer durations were found for spending more time outdoors (420.8 ± 420.8 vs. 355.7 ± 395.1 min/week), training physical strength (117.4 ± 147.3 vs. 86.6 ± 72.7 min/week), sat less (6.74 ± 3.9 vs. 7.1 ± 3 h/day), or practicing other sports (240.3 ± 248.7 vs. 112.7 ± 85 min/week). At the same time, this group used more anthroposophical medicine applications (78.8 ± 167.7 vs. 44.4 ± 43.1 min/week), nasal rinses (4.7 ± 3.3 vs. 3.8 ± 2.3 use/week), underwent more therapeutic fasting days (14.5 ± 17.8 vs. 11.8 ± 9.7 days/year), and conducted hydrotherapy or water treatments for shorter time periods (51.3 ± 74.8 vs. 65.9 ± 57.2 min/week), digital health applications (956.5 ± 4,004 vs. 1,048.8 ± 2,318.7 min/week) and aromatherapy (330.1 ± 769.2 vs. 655 ± 1,132.4 min/week).

In terms of use of CIM self-help and lifestyle interventions, there were no major differences in COVID-19 participants compared to the overall sample. A slightly larger proportion of the COVID-19 participants reported a negative emotional state (8.1%; *n* = 5) compared to the overall sample (7.6%; *n* = 87).

Regarding naturopathic remedies (including herbal remedies or supplements, anthroposophical or homeopathic drugs) *n* = 718 (63.1%) took at least 1 remedy, *n* = 537 (47.2%) took 3–5 remedies, and *n* = 185 (16.3%) took 6 or more remedies.

### 3.6. Diet during the COVID-19 pandemic

More than half of participants followed a plant-based diet and stated to be vegetarians or vegans (56.3%; *n* = 640)–the majority of these followed a vegan diet (32.1%; *n* = 365), which excluding all animal products, or a lacto-ovo-vegetarian diet (15.6%; *n* = 177), excluding animal products except eggs and dairy (Supplementary Table 5). One-third of the overall sample followed an omnivorous diet (32.7%; *n* = 372), including both plant and animal food.

Regarding gender differences, male subjects were slightly more likely to follow an omnivorous diet than women (+4.9%). Other diets, such as those based on Traditional Chinese Medicine or Ayurvedic principles, played a subordinate role (Supplementary Table 5).

Concerning the differences in the different age groups, a higher proportion of participants younger than 30 years was found to follow a vegan diet (18–30 years: 55.3%; *n* = 73) compared to older ones (31–50 years: 33%, *n* = 133; 51–65 years: 28.5%, *n* = 140; ≥66 years: 17.1%, *n* = 19). Accordingly, the older ones were more likely to follow an omnivorous diet (31–50 years: 29%, *n* = 117; 51–65 years: 37.2%, *n* = 183; ≥66 years: 45%, *n* = 50) compared to younger ones (18–30 years: 16.7%; *n* = 22).

Interestingly, participants in a positive emotional state tended to follow a vegan diet (+11.1%) more often (32.9%; *n* = 190) than participants in a negative emotional state (21.8%; *n* = 19). Moreover, participants in a positive emotional state reported a higher proportion of organically grown products in their diet (69.7 ± 23.8%) compared to participants in a negative emotional state (57.8 ± 26.7%) and an overall healthier diet with an emphasis on plant-based and less processed foods.

No major differences in dietary patterns were found between subjects with the respiratory diseases mentioned above and the overall sample.

### 3.7. Self-efficacy (General Self-Efficacy Short Scale, GSE-3)

The overall sample achieved a mean scale value of 4.1 ± 0.6 in the assessment of general self-efficacy, with the maximum achievable mean scale value being five (Supplementary Table 6). There were no major differences regarding gender and different age groups. In addition, participants in a negative emotional state showed lower levels of general self-efficacy (3.8 ± 0.8) than participants in a positive emotional state (4.3 ± 0.5). No major differences in general self-efficacy scores were found between subjects with the respiratory diseases mentioned above and the overall sample.

### 3.8. Well-being (World Health Organization Well-Being Index, WHO-5)

The overall sample achieved a mean scale value of 15.2 ± 5 in the assessment of wellbeing, with the maximum achievable mean scale value being 25 (Supplementary Table 6). The following differences were found in the level of wellbeing: males showed higher levels of wellbeing (16.3 ± 4.5) than females (15 ± 5.1). Subjects younger than 30 achieved a lower wellbeing index (18–30 years: 14.1 ± 4.5) than older respondents (31–50 years: 14.7 ± 5; 51–65 years: 15.6 ± 5; ≥66 years: 16.8 ± 5.1). Furthermore, subjects in a positive mental/emotional state showed a higher wellbeing index (17.4 ± 4) compared to subjects in a negative emotional state (9.5 ± 5).

Participants who reported COVID-19, influenza, and other respiratory diseases achieved a lower wellbeing index (COVID-19: 14.6 ± 4.7; influenza: 12.3 ± 6.3; other respiratory diseases: 14 ± 5.2) compared to the overall sample.

### 3.9. Mental/emotional state

Only low scores were reported on average for mental/emotional state during the COVID-19 pandemic (Supplementary Table 6). Of the eight questions (NRS 0–10) regarding mental/emotional state the item “negative societal consequences (e.g., loneliness, increase in social inequality, political decisions) from the SARS-CoV-2 pandemic” was rated the worst (5.1 ± 3.1) and the item “anxiety since the SARS-CoV-2 pandemic began in Europe (approximately March 2020)” was rated the lowest (2.7 ± 2.5), for the other questions see Supplementary Table 6.

## 4. Discussion

Respondents of this cross-sectional online study regarding self-care and lifestyle interventions during COVID-19 pandemic were predominantly female, middle-aged, and had higher levels of education. Of the participants who had tested positive for SARS-CoV-2 or had had symptoms of COVID-19, none required hospitalization. Use of self-care CIM interventions was high in this population during the pandemic. Respondents used a wide range of CIM self-care methods in addition to the commonly recommended healthy lifestyle interventions. Spending time outdoors, physical activity, naturopathic remedies, healthy dietary patterns, and MBM interventions were favored as individual health promotion strategies. No differences in the use of CIM interventions, dietary patterns, or lifestyle interventions were observed between participants who reported respiratory diseases, including COVID-19, and the overall sample. Only 87 participants had a low mental/emotional state regarding the COVID-19 pandemic.

The sociodemographic characteristics of the study population (predominantly female, higher education, and middle age) are consistent with other studies that examined the characteristics of CIM interventions in the general population (23–25). Our study population appears to be healthier and more health-conscious than the general population in Germany that have higher rates of smoking (20%) and greater rate of obesity (16%) (26, 27). Moreover, our study population had a lower rate of chronic cardiovascular diseases (9%) and chronic respiratory diseases (10%).

Preliminary evidence showed that a healthy diet could reduce the burden of infectious diseases (28–30). In a recent survey among 592.571 UK and US participants, a diet characterized by healthy plant foods was associated with a lower risk (hazard ratio [HR] 0.91; 95% confidence interval [CI] 0.88–0.94) and severe COVID-19 (HR 0.59; 95% CI 0.47–0.74) (29). For immune system functioning and cytokine release, phytochemicals—e.g., from plant-based food—rich of trace elements (zinc, copper, selenium, and iron), vitamins (A, B6, B12, C, D, and E, and folate), docosahexaenoic/eicosapentaenoic acid play key roles in immune system function (30, 31). In our study, participants in a positive mental/emotional state tended to follow a plant-based diet more often. Moreover, they reported a higher proportion of plant-based and organically grown products than participants in a negative mental/emotional state. However, no differences in dietary behavior were observed between participants who reported respiratory diseases, including COVID-19, and the overall sample.

At the time the study was conducted, vaccines against SARS-CoV-2 were rarely available. Individuals regularly using CIM often have rather critical opinions regarding vaccines in general (32, 33). This was also reflected in our study population with 40% of all participants not planning to get COVID-19 vaccinated. However, in spring 2021, first vaccines were launched and few long-term data regarding potential adverse effects of vaccines were available. Presumably, these attitudes might have changed. However, in a recent (December 2021), representative survey commissioned by the German Association of Pharmaceutical Manufacturers (Bundesverband der Arzneimittel-Hersteller, BAH) showed no correlation between vaccination rate and homeopathy use (34). The study sample showed a high level of adherence to COVID-19 regulations on average comparable to the general German population: In September 2020, 88% of the German general population reported wearing a face mask, 89%, adhered hygiene regulations, and 67% reduced social contact (35).

Overall, half of our study participants were found to be in a positive mental/emotional state and only few (8%) participants were in a negative mental/emotional state. Participants with a positive mental/emotional state used CIM interventions on average more and longer than participants with a negative mental/emotional state. In a recent study the use of self-care strategies to prevent COVID-19 and the consultation with health care providers were positively associated with concern about being infected with COVID-19 (18). However, these aspects were not covered in our survey.

The COVID-19 pandemic is associated with higher levels of psychological distress and mental health problems, and particularly the presence of chronic diseases was associated with anxiety and stress (36–40). Compared with the overall study sample, participants infected with other respiratory diseases were on average in a lower mental/emotional state and reported higher levels of psychological stress parameters related to the SARS-CoV-2 pandemic.

The study population showed similar levels of general self-efficacy as a sample representative of the resident population in Germany over the age of 18 (4 ± 0.7) (41). Participants in a negative mental/emotional state showed lower levels of general self-efficacy than participants in a positive mental/emotional state. There is a relationship between low self-efficacy and low mental/emotional state and depression (42). Further research is needed, particularly on how CIM interventions may positively influence self-efficacy.

The level of the WHO-5 is slightly below the level of wellbeing in a sample representative of the resident population in Germany aged 41–60 years (17.5 ± 4.9) (43). Younger participants had lower wellbeing than older participants. Other studies also found an age gradient in which younger participants had worse mental wellbeing than older participants (44). The values for the age groups are below the level of wellbeing in a sample representative of the resident population in Germany at the age of ≤40 years (18.4 ± 4.8) and similar to those of the ≥61-year-olds (16.7 ± 5.1) (43). One may speculate that the consequences of the pandemic restricted the public life of the younger population and thus reduced general wellbeing (45).

In other studies, lower stress was associated with mindfulness (39, 40), which was not directly surveyed in our survey. However, our study population practiced yoga and meditation–presumably also the other queried MBM techniques–at a higher rate than in the general population on a whole (46, 47). This may have effects for mental/emotional state that should be explored further. Moreover, regular times spent outdoors by 90% of our participants could have important effects on the general positive mental/emotional state found here (48). In addition, it is interesting to note that participants reporting COVID-19, influenza, and other respiratory diseases achieved only a slightly lower wellbeing index than the overall sample.

Several studies investigated lifestyle changes during the COVID-19 pandemic, e.g., exercise, nutrition, and sleep patterns (19). In an online survey among the general population living in Spain during the COVID-19 home-isolation, a substantial proportion of participants reported meaningful lifestyle changes during the COVID-19 pandemic (19). Most participants reported substantial changes on time spent outdoor (94%) and physical activity (70%). Moreover, about one third of participants reported significant changes on stress management, social support, and restorative sleep (19). In another survey with a total of 338 adults, 68.8% indicated that they participated in mind-body activities during the early months of the COVID-19 pandemic (20). Physical activity was the most frequently (61.5%, *n* = 227) used practice, followed by meditation (*n* = 221), breathing techniques (*n* = 229), and relaxation techniques (*n* = 213). In this study, commonly cited reasons for using mindfulness practices were to promote health, reduce stress, and relax.

The strengths of our study lies in its relatively large sample-size, including respondents from all over Germany, so that more general assumptions can be made about tendencies in the German population using CIM strategies for health promotion. Also, we included validated instruments and different non-profit organizations in the spread of our survey, so that different types of CIM users could be reached.

Several limitations of our study need mention. First, a cross-sectional survey is unable to confirm a direct causal relationship between healthy self-care/lifestyle CIM interventions and COVID-19 risk nor can specific mechanisms be identified. Second, our study population is not a representative or random sample of the general German population; our study population is more a profile of the typical CIM user (majority female, well-educated, practicing a healthy lifestyle). Third, our results may have a bias because of the long data collection period, which, included both the first and second wave of the COVID-19 pandemic in Germany. Also, we did not assess lifestyle changes, and depended on reliable values and data entry. The prevalence of COVID-19 was variable during the long recruitment period and could have affected internal validity. First evidence suggests that lifestyle/dietary changes may be altered in both negative and positive ways during lockdowns (49, 50). Fourth, the self-reported nature of the survey is prone to measurement error and bias. Fifth, a survey of CIM use prior to the pandemic would have been of interest for comparison of CIM use during the pandemic. Finally, data on further comorbidities (e.g., diabetes) were not collected in this study, which may limit the results. Linkage with general practice data on comorbidities would strengthen future research (51, 52).

## 5. Conclusion

Complementary and Integrative Medicine self-care strategies and practices, including general lifestyle interventions, as spending time outdoors, healthy eating, physical activity, naturopathic remedies, and MBM exercises were practiced most frequently during the COVID-19 pandemic by this study responders. Study participants with a positive mental/emotional state used CIM interventions including lifestyle interventions more frequently and for a longer time on average than participants with a negative mental/emotional state. Further research, preferably studies including a control group using a representative sample should further clarify the impacts of the use of CIM self-care strategies on health and on the mental/emotional state. Also, follow-up studies are needed to determine whether the use of lifestyle interventions change over the course of the pandemic and how different lifestyle factors may influence susceptibility to and progression of COVID-19 as well as manifestation of Long- and Post-COVID symptomatology.

## Data availability statement

The raw data supporting the conclusions of this article will be made available by the authors, without undue reservation.

## Ethics statement

The studies involving human participants were reviewed and approved by Ethics Committee Charité – Universitätsmedizin Berlin. The patients/participants provided their written informed consent to participate in this study.

## Author contributions

MJ, GS, HC, and CK: methodology, project administration, and conceptualization. MJ and AE: data curation and writing—original draft. FK: formal analysis. MJ: visualization, investigation, and software. MJ, BB, AM, GS, HC, and CK: supervision. DK, MO, LJ, BS, GR, SB, BB, AM, GS, HC, FK, and CK: writing—review and editing. All authors contributed to the article and approved the submitted version.
